# The Price of Becoming a City: Decentralization and Air Pollution—The Evidence from the Policy of County-to-City Upgrade in China

**DOI:** 10.3390/ijerph192315621

**Published:** 2022-11-24

**Authors:** Zhihong Zeng, Chen You

**Affiliations:** 1 School of Public Administration, Hangzhou Normal University, Hangzhou 311121, China; 2 School of Public Affairs, Zhejiang University, Hangzhou 310058, China

**Keywords:** county-to-city upgrade, decentralization, air pollution, satellite data

## Abstract

It is necessary to reassess the pollution effects of decentralization reforms to improve the future policy design for better economic and social development in the postepidemic era. This study examines the relationship between decentralization and air pollution by exploiting the policy of County-to-City Upgrade in China from 2005 to 2018. Upgrading empowered new cities in fiscal, administrative, and economic matters without changing the political hierarchy. Under the cadre evaluation system, the new county-level city government preferred to increase construction land area and attract more polluting firms to promote economic development, and air pollution became more severe. Heterogeneity tests found that when the new city was located in eastern China or was away from the provincial boundary, decentralization would induce more severe air pollution. Overall, decentralization without a supplementary incentive rule has a negative effect on air pollution.

## 1. Introduction

The fallout from the winter 2019 outbreak of COVID-19 on the global economy is still lingering. On the one hand, COVID-19 endangers human health, affects the reproduction of human resources and labor supply, weakens consumer demand, and leads to pessimistic expectations; on the other hand, the lockdown policy in many countries disrupts the social production order, hits the global supply chain system, inhibits economic and social development, and leads to slow global economic growth and recession in some countries. Therefore, how to use feasible policy tools to restimulate economic growth out of the recession has become a central topic of concern in practice and academia. While decentralization reform is an important policy tool for policy makers, it may cause potential environmental pollution problems while developing the economy, making economic recovery in the postepidemic era risky. Thus, it is necessary to reassess the pollution effects of decentralization reforms to improve the future policy design for better economic and social development in the postepidemic era.

The impact of decentralization on environmental pollution is still inconclusive. A fortiori, both proponents and opponents have corresponding evidence to support their respective views [1]. Whether decentralization can improve or degrade the environment is still a controversial question to answer. To fill those gaps, this paper wants to use a new institution background to assess the effect of decentralization on air pollution and its mechanism.

Therefore, this study exploits the policy of County-to-City Upgrade (CCU) (che xian she shi) in China to examine the effect of decentralization on air pollution. Drawing on the DID approach, we found that air pollution increased after the CCU. This paper measures air pollution by using satellite images [2]. The DID estimation results implied that the mean of air pollution increased by 4.04 during the sample year. Then, we investigated the channel behind the connection between the policy and air pollution. The key mechanism is that the new city government will increase construction land area and attract more polluting firms to promote economic development. Our findings showed that the construction land area increased by about 165.7 km^2^, and about ten air-polluting firms increased. Moreover, the location of the new city makes a difference in air pollution. The air pollution will worsen if the new city is in eastern China. However, the in-provincial city significantly differs compared with the provincial boundary sample. One possible explanation is that provincial boundary counties often have a spillover effect [3], and the deterioration is not prominent.

Although the central government has the final decision power to decide which county could obtain the policy, a key challenge is that the reformed counties are not randomly selected. To tackle this concern, we controlled the main determinants in the selection. Beyond that, we used an event study to estimate the pre-trend assumption. Additionally, we made use of the sample for the treatment group. Finally, we constructed a placebo test by randomly assigning treatment status to counties [4].

The remainder of this paper is organized as follows: Section 2 is a literature review. Section 3 provides the policy of the CCU background and theoretical mechanisms. Section 4 presents the data and estimation framework in detail. Section 5 describes the main empirical findings and robustness checks. We explore the underlying mechanisms and heterogeneous effects, respectively, in Section 6 and Section 7. Section 8 discusses our contribution, and Section 9 concludes this study.

## 2. Literature Review

According to the classic decentralization theorem, local governments could improve social welfare within a jurisdiction [5]. Therefore, the devolution of power to local governments can induce better environmental outcomes. In detail, the mobility of labor, capital, and residents drives local governments to compete, i.e., “vote by foot” [6,7], and decentralization demands that local governments meet the preferences of residents based on information advantages and are directly accountable to the residents [8,9]. Furthermore, higher-level governments can use Yardstick Competition to assess whether local governments meet residents’ preferences [10]. However, other scholars doubt the effectiveness of decentralization. First, there are many assumptions underlying the “vote by foot” model and its extensions, such as constraints on residential migration, the existence of spillovers, and the availability of a sufficient number of local governments [11]; additionally, rational voting behavior is a crucial assumption of the model [12,13]. Second, many factors influence whether downward decentralization is effective, such as accountability systems [14,15], political party factors [16,17], etc. Finally, decentralization may also lead to “race to the bottom” [18] and the emergence of being captured by local elites [19], resulting in decentralization failing to achieve the desired effects [20]. In other words, decentralization may interact with other political factors leading to controversial consequences.

In general, fiscal decentralization and environmental federalism are two primary representative types in the decentralization literature. In fiscal decentralization, some cross-country comparative studies argue that fiscal decentralization reduces carbon dioxide emissions [21,22]. In specific country studies, fiscal decentralization in Pakistan reduced carbon dioxide emissions from 1984 to 2018 [23], while fiscal decentralization in the United States made it difficult for local governments to cope with natural disasters [24]. Some assert that fiscal decentralization can motivate the local government to increase pollution abatement spending and take advantage of local information [25,26]. Nevertheless, others claimed it might lead to a “race to the bottom” between local governments. From this perspective, local officials may compete for local tax resources to promote economic growth by easing regulation [27,28]. Overall, on the one hand, existing studies suggest that fiscal decentralization has worsened environmental quality [29,30]. However, the other side of the literature argues that fiscal decentralization improves the environmental quality [31,32].

In environmental federalism, this category of literature analyzes how authority related to environmental governance is divided among different levels of government, and some studies argue that environmental decentralization is beneficial to environmental governance [33,34]. Similar to the implicit assumptions of traditional decentralization theory, the effectiveness of environmental federalism depends on the corresponding preconditions: (1) the absence of externalities; (2) the existence of many jurisdictions; (3) the availability of all economic rents to competing local jurisdictions; (4) welfare-maximizing local regulators; (5) the absence of restrictions on feasible policy instruments; and (6) the absence of redistributive policies [31] and the setting of specific model assumptions, such as the presence of environmental pollution spillover, heterogeneity of local environmental preferences, and other parameters to discuss the effects of decentralization in environmental governance [35]. On the one hand, proponents claim that local policymakers can acquire more information and respond better to local people in environmental governance [36,37]. On the other hand, inter−jurisdictional spillover and “race to the bottom” fundamentally challenged environmental federalism [1]. The effectiveness of decentralization remains debatable in the literature [38].

In sum, despite two controversial studies where both have the theoretical framework and empirical proof, there is not enough evidence on how decentralization affects environmental quality. Firstly, it is not easy to have a causal interpretation since decentralization or centralization usually coexist with some exogenous variation [39]. Secondly, two contrary findings indicated that some assuming condition is crucial [28]. Thirdly, some called for more research on how local institutional factors influence the effectiveness of decentralization in developing countries [40].

Therefore, this study examines the relationship between decentralization and pollution in the context of the CCU. To the best of our knowledge, existing studies neglect to use this policy to analyze the relationship between decentralization and environmental performance, so this paper supplements this lacuna. There is a nested multitier administrative level (province–prefecture–county–town) in China’s administrative hierarchy [41]. At the county level, city, county, and district often have the same administrative rank but differ in power distribution. After the CCU, the new city retains the same administrative rank but has more power in many fields [42,43]. Therefore, this policy provides an especially advantageous context to analyze how decentralization influences the environment.

## 3. Institution Background and Theoretical Analysis

### 3.1. The Policy of County-to-City Upgrade in China

China’s contemporary administrative structure is organized in four levels, and a fundamental feature is a decentralized economy within centralized politics. Each level has different power in fiscal, administrative, and economic matters. The power often decreases with the downward level. However, there is diversification in power distribution at the same level, such as at the city, county, and district at the county level. In each administrative division, local officials are mainly accountable to their superiors (province–prefecture–county–town), constituting a multitier and vertical administrative system [44]. Upper-level officials usually assess their subordinates through many criteria, and economic development is the most crucial. Thus, every local official is incentivized to try his/her best to promote local economic development [45,46], and a common way is obtaining more power on local affairs such as administrative, personnel, and fiscal power [47]. At the county level, many counties want to become a county-level city to obtain more decision-making power to compete with other counties. Table 1 describes the potential benefits of becoming a county-level city.

**Figure 1:**
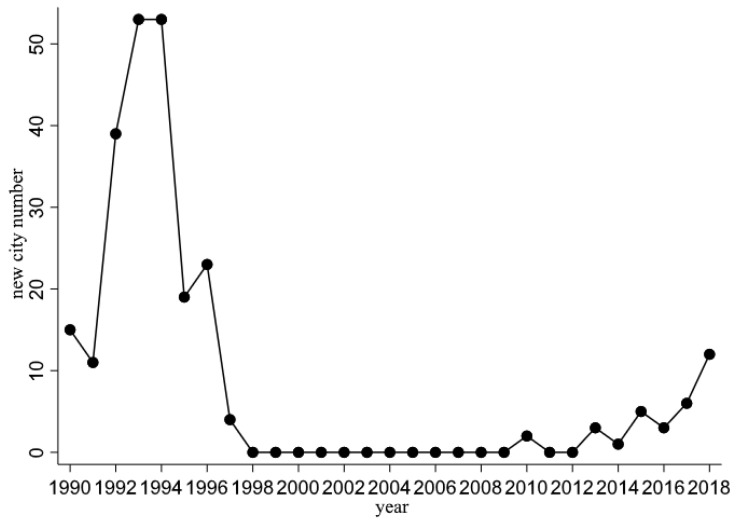
The number of new cities during 1990–2018. Note: From 1998 to 2009, the central government had paused applications for county-level cities. The approval procedure did not reopen until 2010. Data source: the website of the Ministry of Civil Affairs. Data source: 2014 Hangzhou Statistical Yearbook.

**Figure 2:**
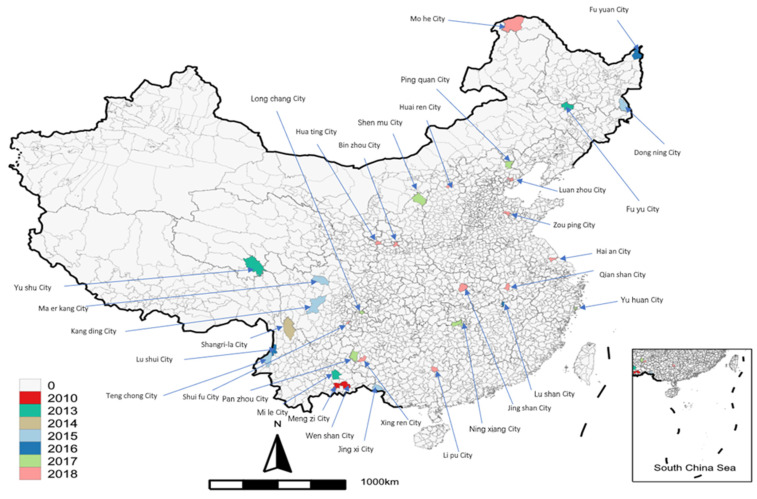
The location of new cities during 2005–2018. Data source: the website of the Ministry of Civil Affairs.

In fact, the central government had used this policy before 2000 and published a detailed requirement for the CCU in 1993 [52]. However, the central government realized this policy had many adverse effects on urbanization, land use, and fixed investment [43]. As a result, the approval procedure was stopped after 1997. For a long time, the central government only ratified a few new cities; after 2016, the new approved number constantly increased (Figure 1). Most new county-level cities are inland, and a province is usually expected to approve only one new city in a year (Figure 2).

In 2016, the central government and the Ministry of Civil Affairs published two documents on applying standards and approval procedures. Although the original paper is not publicly available, we could obtain the primary information from some government websites (We obtained this information from some websites such as: http://news.hsw.cn/system/2020/0806/1220562.shtml; https://sichuan.scol.com.cn/ggxw/201908/57037218_2.html; https://user.guancha.cn/main/content?id=43089, last accessed on 1 November 2022). The four primary standards were urbanization rate and population number, economic index, municipal facilities, and essential public services requirement. Significantly, many economic indexes are supposed to satisfy the standard for two consecutive years. In principle, each provincial government can only submit a new application until the latest one is finished. However, this policy lacks supplementary restrictions on environmental protection. Unlike economic and social targets, the CCU policy does not include enough environmental standards that the applicant should meet.

### 3.2. The Pro–growth Strategy in Construction Land and Firm Settlement

In China, political power is centralized upward. Moreover, whether the local government leader (county party secretary or county magistrates) can obtain a promotion is mainly decided by their superior. They need to compete with their peers in neighboring jurisdictions in this cadre evaluation system. Therefore, they have the incentive to develop a local economy to stand better chances of promotion because the economic index still is the most important factor in political assessments [46]. Consequently, in the CCU, the policy endows more power to a new city but with insufficiently detailed restriction rules. It suggested that local officials have more independent power without supplementary incentive rules and will prefer to adopt a pro–growth strategy to boost the local economy. Meanwhile, air pollution becomes more severe if the local government leader only considers their economic target.

In practice, the first pro–growth strategy is obtaining more construction land for economic development. Not only can the construction land be used to build infrastructure, for example, roads, bridges, and hospitals, but it is also a crucial resource for local governments to influence the local economy and obtain more revenue [51]. According to the CCU policy, the provincial or prefecture-level government will prioritize the new county-level city in a construction land quota distribution and will permit them to keep a high percentage in land sale revenue. Additionally, in China, the government controls most of the land supply, and the new county-level city can obtain more power in deciding when and how to use or sell construction lands. Therefore, on the one hand, the more construction lands the new county-level city has, the more firms could relocate. On the other hand, they could sell construction lands at a high price to subsidize building infrastructure. In general, after the CCU policy, the new county-level city will take advantage of the power in construction land to increase local economic growth, and in the process of turning farmland into urban construction land or building new factories and apartments on construction land, these human behaviors will cause air pollution [54].

The second pro–growth strategy is attracting more firms, including air polluting factories. Firms not only bring job opportunities and increase local GDP, but also discharge pollutants, especially in some manufacturing industries [44]. On most occasions, counties lack the sufficient power in industrial planning and bid for investments and need to obey investment cap restrictions. Thanks to the CCU policy, the county-level city gains more independence in the economic matter, such as approving investment projects with a higher investment cap, designing industrial planning without more limitations, etc. Consequently, the county-level city government can compete with other counties for investment projects and new firm settlements. To increase revenue and local GDP, the county-level city government may attract some air-polluting factories. These companies often belong to the intensive capital industrial sector and can contribute to the local economy. Therefore, air pollution is inevitable to some extent.

## 4. Estimation Methods

### 4.1. Estimation Framework

**Figure 3:**
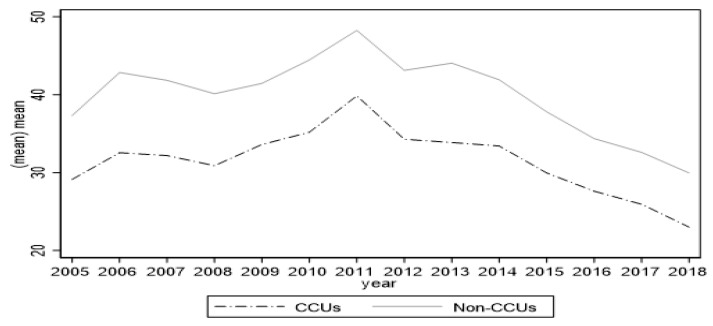
Air pollution trend comparison 2005–2018. Note: the figure illustrates the time trends of PM2.5 (mean) of the CCU counties (i.e., counties that adopted the CCU policy since 2005) and that of non-CCU counties (i.e., counties that did not adopt the CCU policy during the sample period).

As the CCU policy is only adopted in some counties and the number of those counties changes every year, it is suitable to use the difference-in-differences (DID) estimation. DID estimation involves comparing county variation and time variation in the same period. Figure 3 illustrates the similar trends during our sample year, hence following previous research [4,49]. The baseline DID specification is Equation (1):

(1) $$Y_{ct}=\beta_{0}+\beta_{1}Policy_{ct}+\delta Control_{ct}+\alpha_{c}+\gamma_{t}+\varepsilon_{ct}$$

where *c* and *t* represent county and year, respectively; $Y_{ct}$ indicates the mean of PM2.5 for county *c* in year *t*; $Control_{ct}$ is a vector of time-variant control variables, including some applying standards; $\alpha_{c}$ and $\gamma_{t}$ are the county fixed effect and year fixed effect, respectively; $\varepsilon_{ct}$ is the error term; and $Policy_{ct}$ is the regressor of interest. Specifically, $Policy_{ct}=treatment_{c}•\;Post_{ct}$ if county c was reformed during the sample period, $treatment_{c}=1$ and 0 otherwise. $\;Post_{ct}$ is assigned value of 1 if $t\;\geq \;t_{c0}$, where $t_{c0}$ is the year county c carried out the CCU policy, and 0 otherwise. We clustered the standard errors at the county level to solve the potential serial correlation and heteroskedasticity problems.

### 4.2. Data and Variables

The data contain socioeconomic indicators and air pollution at the county level in China. As Figure 1 illustrates, in 2010, the approval procedure reopened and the CCU policy began, and our air pollution data ended in 2018. As a regular practice, the start of the sample period is expected to be several years earlier than the time when the policy is adopted. Here, our panel data just cover 2005–2018. 

The dependent variable is county-level air pollution data. We use PM2.5 as the measurement for air pollution (PM2.5 data are from: https://sites.wustl.edu/acag/datasets/surface-pm2-5/#V4.CH.03 last accessed in 10 December 2020), which has been commonly applied by recent articles such as Hammer et al. [55]. These are the annual average concentration data, covering 2005–2018, and most of them are incorporated with a 0.01° × 0.01° resolution (about 1 km by 1 km). We extracted the PM2.5 data from satellite images by using ArcGIS. 

The year when the policy began in each county was obtained from the Ministry of Civil Affairs website. To solve the problem of county-level administrative changes, some counties were excluded if their administrative boundaries had changed in the sample year and the administrative division code was applied to code each observation, since the code is assigned by the Ministry of Civil Affairs and is unique for each administrative division. In addition, some observations concerning four municipalities were dropped out because the counties or districts they administer have a higher administrative rank. Additionally, we used an administrative division code to calculate whether the city/county belongs to a sub−province city.

We manually collected socioeconomic data from the *China County Statistical Yearbook* and supplemented the data with the help of annual statistical yearbooks of many provinces. The construction land data were derived from CSMAR during 2005–2016. The original data recorded every land transaction in detail, and we classified the data of the construction land at the county level to match the corresponding county/city. Moreover, following Cai et al. [27], we used “Report on the First National Census of Polluting Sources” and “Report on the Second National Census of Polluting Sources” (The original report is from the Ministry of Ecology and environment’s website (before 2018 its name was the Ministry of Environmental Protection)) to identify eleven major air polluting industry sectors and unify industries sector codes to calculate the number of polluting firms in every county/city (Because in 2011 NBS used new industry sector codes, we revised the industry sector code according to the 2011 version. The eleven major air polluting industries are: coal mining and washing (06), power and heat production and supply (44), nonmental mineral production (30), ferrous metal smelting and rolling processing (31), chemical raw materials and products manufacturing (26), petroleum, coal, and other fuel processing (25), rubber and plastic products (29), nonferrous metal smelting and rolling processing (32), papermaking and paper products (22), wood processing and products (13), and agricultural food processing (20)). The dataset came from the *Annual Survey of Industrial Firms (ASIF) released by the National Bureau of Statistics (NBS) (2005–2013)* (The credible ASIF dataset is from 1998 to 2013 and most studies use it). The ASIF has been used in previous studies and provides basic firm information (name, address, industry sector, etc.) [27].

Location data is a dummy variable used to measure whether a county-level administrative division is in provincial boundaries or to determine which region of China it belongs to. Moreover, we used ArcGIS to calculate the distance between counties/cities and their prefecture-level city. Table 2 provides a brief description of all the data above.

### 4.3. Pre–trend Test

The DID method requires no significant difference between the treatment and control group before the policy begins. Without meeting the implicit assumption, the estimation results are incredible. Therefore, based on the event study, following the pre−trend test method [4], the estimation of the year-wise change in air pollution was constructed to verify the assumptions. We revised the independent variable of Equation (1) and then obtained Equation (2); the others are the same:

(2) $$Y_{ct}=\beta_{0}+\beta_{k}\sum_{k>-4}^{5}D_{ct}^{k}+\delta Control_{ct}+\alpha_{c}+\gamma_{t}+\varepsilon_{ct}$$

The dummy variables $D_{ct}^{k}$ represent a window of policy events. $t_{c}$ is the year when the county c adopts the CCU policy and t is the year variable (from 2005 to 2018). $D_{ct}^{k}$ equals 1 if $t-t_{c}=k,\;k\in \left(-3,-2,-1,0,1,2,3,4,5,5+\right)$ and county c is the treatment group; otherwise, it equals 0. The omitted time category is k≤−4. Because many economic indexes need to meet the standard for two consecutive years, we chose k = −4 as the baseline year. If the coefficients of Equation (2) are not significant when k≤0, it suggests that the CCUs and non-CCUs followed similar time trends before the CCU policy.

**Figure 4:**
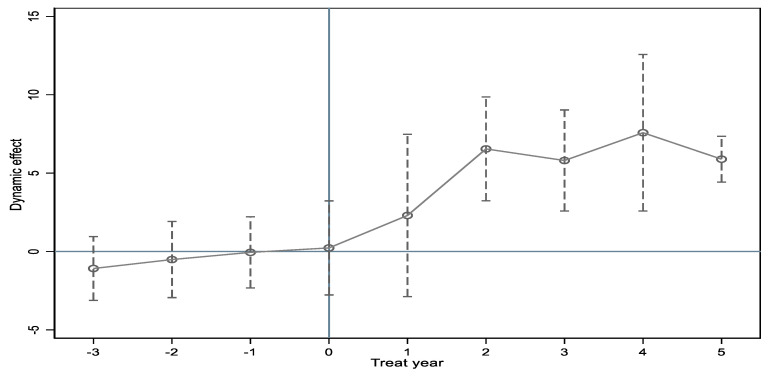
The pre−trend test of the CCU policy.

The coefficients of Equation (2) are shown in Figure 4. The confidence interval is 95%, and the result concludes that Equation (1) is consistent with the pre−trend test. 

## 5. Main Results

### 5.1. Baseline Estimation

Table 3 presents the empirical results of Equation (1). Column (1) and column (2) both show a positive and statistically significant effect relationship between the policy and air pollution. Column (2) reveals that the coefficient is 4.04. This outcome implies that decentralization may have a negative effect on local air pollution.

### 5.2. Robustness Checks

#### 5.2.1. Alternative Reform Policy Interference

The baseline estimation might include confounding factors if other related policies were implemented during the sample period. Then, the outcome may be untrustworthy. In addition to the CCU policy, the county (city)-to-district reform (xian shi gai qu) also changed China’s administrative structure. Contrary to the CCU, that policy is a centralization reform and makes local officials more dependent on their superiors. For example, the new district will lose its approval powers on land use, infrastructure construction, and development plan [56]. To address the potential concern, we eliminated the sample that had this reform during the same period. The result is shown in column (1) in Table 4. The outcome still supports the previous conclusion. 

#### 5.2.2. Alternative Subsample

In the sample period, the CCU involved different reforming counties yearly, providing rich variation. All samples were used in the baseline estimation process and the samples were assumed to be more homogeneous. Hence, the reformed counties were chosen in the robustness check to verify the previous result. The same strategy was also used by previous studies [4].

The results are reported in column (2) in Table 4 and are similar to baseline estimation in terms of statistical significance.

#### 5.2.3. Alternative Administrative Structure Impact

In addition to the four levels of the administrative structure, some prefecture-level cities have vice-provincial status. Most of them are the capitals of their provinces or some essential cities. The status will grant those cities some power on socioeconomic affairs, which equals the provincial level. Furthermore, the counties or districts administered by vice-provincial cities may have a higher administrative rank and more power. For example, in Hangzhou city, the street may have a county-level administrative rank, but the street only has town-level status in most other cities. Therefore, we excluded those samples in the estimation, and the result is reported in column (3) in Table 4. The coefficient had a similar positive effect and statistical significance.

#### 5.2.4. Clustering the Standard Errors along Two Dimensions

According to other research [3], considering the series correlation, we clustered by county. Meanwhile, to handle potential problems of heteroskedasticity and spatial correlation, we conducted a double cluster standard error by county/year in column (4) in Table 4. Fortunately, the coefficient is the same as in column (2) in Table 3 and the standard errors just changed a little.

### 5.3. Placebo Test

Even though the baseline estimation had controlled many applied standards, the results may still have been influenced by any omitted variable. Thence, a placebo test was conducted by randomly assigning the CCU policy to counties (for similar practices [4,57]). Specifically, in our sample period, the CCU policy only took effect seven times {2010(1), 2013(3),2014(1), 2015(5), 2016(3), 2017(6), 2018(12)}. There should be a significant difference between the coefficients of the baseline estimation discussed in part 4.1 and the 500 times random sampling estimation discussed here. In addition, the coefficient of the random sampling result should be close to zero; otherwise, it would imply that a mistake occurred in the DID estimation. We repeated the randomization 500 times to avoid contamination and to improve the statistical effect of this placebo test.

**Figure 5:**
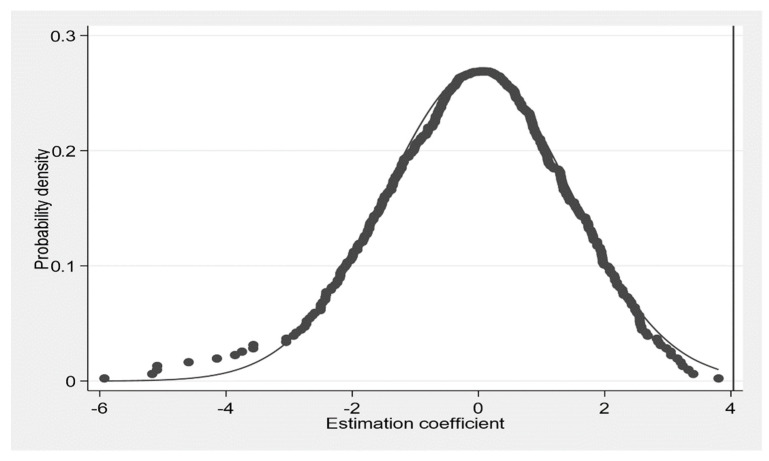
Distribution of 500 estimations using randomization data. Note: the vertical line stands for the outcome of column 2 in Table 3.

Figure 5 shows the distribution of the 500 estimation results of the 500 times random sampling and the baseline estimate, 4.04, shown in column (2) in Table 3. Most of the random samplings’ estimation results were close to zero and the baseline estimate clearly lied outside the entire randomization outcome. Altogether, these results imply that the omitted variables did not severely bias our baseline estimation.

### 5.4. Spillover Effect Test

Air pollution often has a spillover effect and is associated with industrialization and population agglomeration. Moreover, the geographical distance will influence the intensity of the spillover effect. Hence, once the new county-level city is close to its prefecture-level city, the latter may affect the former in air pollution. It indicates that our baseline estimation is biased if the spillover effect occurs. To deal with this problem, we excluded the sample if the distance between counties/cities and their prefecture-level city was less than 50 km, 60 km, and 70 km to check the spillover effect. If the coefficient decreases and becomes insignificant, our estimation is untrustworthy; otherwise, the baseline outcome is credible. Table 5 reports the results of excluding geographical distance, and all the outcomes were positive and significant. 

## 6. Underlying Mechanisms

In the previous section, we provided evidence that the new county-level city had worse air pollution after the CCU policy. This section examines the underlying mechanisms behind the CCU policy to strengthen our baseline results. Specifically, we examine whether the CCU policy enhances the construction land area and the number of air-polluting firms.

### 6.1. Effect on Construction Land Area

First, we examined whether the CCU policy affected the construction land area in the new county-level city. After the reform of the tax-sharing system in 1994, local officers have lacked enough power to control and operate state-owned enterprises to promote economic growth. Furthermore, construction land has become an essential resource mastered by local officers to build infrastructure and attract economic factors. Due to the CCU policy, local officers can obtain more construction land quotas from their superiors without a supplemental rule on environmental protection. Thus, the local officers will try their best to increase the construction land area.

Since the construction land area data of counties/cities are not available in an official report or official statistical yearbook, we used data from the CSMAR dataset because it combined detailed land transaction information at the parcel level during 2005–2016. We extracted the construction land data by distinguishing the land-use type and sum parcel-level data to match each counties/cites so the data can represent the number of construction land areas that local officer can use. We estimated Equation (1) by using the construction land area as the dependent variable from 2005 to 2016; the others were the same. The result is shown in column (1) in Table 6. Compared with the unreformed counties, the CCU increased the construction land area by about 165.7 km^2^, suggesting the policy had endowed local officers more power to obtain a construction land quota.

### 6.2. Effect on Air Polluting Firm

Second, we examined whether the CCU policy affected the air-polluting firm in the new county-level city. Industrial firms not only promote the local economy, but they also pollute the local environment, especially in some industrial sectors. For example, petroleum refining firms often emit gas that pollutes the air, but it can bring a considerable investment and provide stable taxation. If local officers need to meet the economic target for promotion, they may prefer to attract those firms. After the CCU, the local officer can approve projects with a higher investment cap and design their economic plan. Therefore, local officers both have the incentive and the ability to attract investments, including from air-polluting firms.

Because the official statistical yearbook just reports the total number of industrial firms on the county level, we used the ASIF dataset (2005–2013) to calculate the number of air-polluting firms in each county/city by the industries sector code. We used Equation (1) and changed the dependent variable to the number of air-polluting firms; the other variables were the same. Column (2) in Table 6 shows a positive and significant effect on the number of air-polluting firms, implying that the policy induced more air-polluting firms in the new county-level city and polluted the air.

## 7. Heterogeneous Effects

Thus far, we have provided evidence that decentralization has a negative effect on air pollution and estimated the average effect of the CCU policy on air pollution. With the information on county/city geographical location, it is feasible to examine the potential heterogeneous effects among sample periods. Specifically, we investigated whether geographical locations have different outcomes because different regions of China are in an unbalance development stage, and air pollution has a strong spillover effect among administrative jurisdictions. Thus, we used Equation (1) to estimate the geographical effect by grouping where the county/city is located in.

### 7.1. Provincial Boundary Effect

We divided the observations into two groups according to whether the county/city was in the provincial boundary. In detail, if the county/city shared the administrative boundary with another province, we defined this county/city as being in the provincial boundary. The provincial boundary hardly ever changed during our sample year, so we extracted the location data from the China map.

The results are represented in column (1) and column (2) in Table 7. Compared with the provincial boundary sample, the in-provincial city had a significant effect. Because provincial boundary counties often have a spillover effect, the deterioration is not prominent. However, once the in-provincial city obtains more power, the air pollution will worsen more than in other counties if they are in the internal of the province.

### 7.2. The Difference between Provinces

We grouped the observation into two subsamples: the eastern region and the midwest region, because if the county/city is located in the eastern region, it indicates that the county/city has a higher industrialization and urbanization rate. Local officers need to invest more resources to compete with their peers in the promotion. Thus, the air pollution is more severe if they obtain more power after the CCU policy.

The estimation results are shown in column (3) and column (4) in Table 7. The outcomes imply that compared with the midwest region, new cities in the eastern region will exert more effects to develop the local economy and worsen the air condition.

## 8. Discussion

It is well known that decentralization reform can promote economic growth and is one of the essential options for countries to revitalize economic development in the postepidemic era. However, the relationship between decentralization and environmental outcomes has not yet reached academic consensus and needs to be further verified to provide possible empirical references for subsequent decentralization reforms. Based on the study of policy reforms in China’s CCU, decentralization reforms without a supplementary incentive rule have caused environmental degradation. That is, decentralization harms the environment. Therefore, in future decentralization reform, a corresponding incentive rule should be set to avoid the negative impact of the reform.

Firstly, this paper complements the enormous existing literature on the environmental outcome of decentralization. Many counties have reformed their political structure in various forms to begin decentralization and, especially in developing countries, have a severe environmental problem [58]. Decentralization often includes a broad array of power—political, regulation, fiscal, and different policies. Therefore, conflicting and inconclusive results often emerge in many empirical studies [59]. By contrast, the policy of the CCU has allocated substantial power in many affairs but kept the same political structure. Thus, this policy can prove that decentralization without a supplementary incentive rule has a negative effect on air pollution. Existing research has investigated many environmental outcomes, including environmental performance and policymaking [3], environmental enforcement [29], local firm behaviors [60], the interaction with other factors [61], and some policy instruments for central government inspection [62]. Many researchers have debated the causal relationship and realized that the trade−off or supplementary policy is essential [39].

Several recent studies have empirically examined the impacts of decentralization on environmental outcomes, but their results have been mixed so far. Wen and Lee [25] found that fiscal decentralization improved firm environmental performance and that the political assessment metrics of local officials are the critical factor. Song et al. [63] found that decentralization induced the water pollution indicators in pilot counties to decrease. Khan et al. [21] produced findings that indicate that fiscal decentralization improves environmental quality. Banzhaf and Chupp [64] provided evidence that the centralized policy outperforms the decentralized policy in US air pollution. We advance the literature in two ways: First, we used the policy of the CCU as a quasi−experiment for decentralization and to detect the mechanism. Second, we took unique satellite image data as the air pollution indicator to decrease the measurement error, following Ghanem and Zhang [65].

Secondly, this study fits into a broad strand of empirical work concerning territorial reform results [66]. Recent years have seen a rapid process in research on the relationship between territorial reforms and their impacts. The impacts include three major excepted consequences: (1) economic efficiency, (2) managerial impacts, and (3) democratic outcomes, and the results are still far from conclusive [67]. Although many existing studies exist in this area, previous research empirically analyzes environmental consequences. To the best of our knowledge, Lipscomb and Mobarak used county splits to examine the impacts on water pollution in Brazilian rivers [68], and the creation of new jurisdictions in Indonesia led to increased deforestation [69]. Additionally, city–county mergers in China can reduce the urban energy intensity [70]. However, this paper deviates from theirs in two ways and provides new evidence on this topic. First, as a proxy measurement of decentralization, the policy of the CCU keeps the same jurisdictional boundary so the socioeconomic data can be coherent in the sample time. Second, Brazil has a federalist political structure, but China is more centered. Thus, local officials have different political restrictions. Third, Lipscomb and Mobarak only surveyed water pollution, and we added new evidence on air pollution in the current study.

## 9. Conclusions

In this study, we examined the effect of decentralization on air pollution. To decrease the potential endogeneity of decentralization, we used the CCU policy to measure decentralization and combined a rich dataset to check the estimation outcome. The DID estimates implied that the mean of the air pollution increased by 4.04 due to the CCU policy, confirming that decentralization has a negative effect on air pollution. The key mechanism is that the new city government will increase construction land area and attract more polluting firms to promote economic development because this policy lacks supplementary incentive rules in environmental protection. Our findings provide evidence that the construction land area increased by about 165.7 km^2^, and about 10 air-polluting firms increased. Moreover, the location of the new city makes a difference in air pollution. If the new city is in eastern China, the air pollution will worsen. However, compared with the provincial boundary sample, the in-provincial city had a significant effect. Because provincial boundary counties often have a spillover effect, the deterioration is not prominent.

This paper advances our understanding of decentralization and environmental outcomes by providing a CCU policy to carefully address the endogeneity problem in decentralization. Meanwhile, we contribute to growing empirical research on this topic of territorial reforms by using data from a developing country to complement the environmental outcomes of territorial reforms.

Our analysis demonstrated that with the absence of a supplementary incentive rule, decentralization has a negative effect on air pollution. Although the new county-level city obtains more independence in many fields without the supplementary incentive rule, the unintended price is more severe air pollution. It means that with the centralized political system, decentralization in economic power may have unintended environmental consequences. These findings yield essential policy insights that decentralization should have supplementary incentive rules and that each part of the institutional environment is interrelated. Policy decision makers should be sensitive to it when they want to recover the economy in the postepidemic era.

## Figures and Tables

**Table 1:** The Potential Benefits of Becoming a County-level City.

Category	Benefits	Source
Administrative power	County-level cities have more authority on economic and social management, such as approving projects with a higher investment cap, having more flexibility when establishing branches of government, having more independence on city development plan, etc.	[48,49]
Fiscal benefits	County-level cities are likely to receive more fiscal subsidies from the provincial government and have more authority on budget plans and designing tax (fee) policy. For example, they collect higher urban construction tax ratios (7%) compared with counties (5%)	[43,50,51]
Land-related	County-level cities are more likely to obtain more quota for converting farmland to urban construction land and could keep a high percentage in land sale revenue	[42,52]
Policy priority	County-level cities are more likely to obtain preferential policies and investment projects	[49,53]
Rank	County-level cities’ secretary is more likely to become a member of the Standing Committee in its superior governments	[42]
Reputation	County-level cities are more likely to obtain more prestige and are more attractive to investors	[48]

Note: given the volatility of Chinese policies, the benefits are continuously changing over time, and the benefits listed are not necessarily practical during the same period; this is an incomplete list summarized based on existing literature.

**Table 2:** Descriptive statistics.

Variable	Non-CCUs		CCUs		Variable Description
Names	Mean	Sd	Mean	Sd	
pp	0	0	0.210	0.408	The abbreviation of $Policy_{ct}$ in Equation (1)
mean	40.00	22.93	31.53	20.99	The mean of PM2.5 (ug/m3)
gdp2	0.423	0.159	0.476	0.183	Added value of secondary in GDP (%)
gdp3	0.350	0.104	0.349	0.133	Added value of tertiary in GDP (%)
pgdp	27,698	47,800	37,013	34,992	Per capita GDP (yuan)
pland	299.4	292.2	258.6	276.6	Per square kilometer (number of people)
prev	1761	3765	2325	2629	The county government fiscal revenue per capita (yuan)
rev	79,627	164,836	109,376	152,596	The county government fiscal revenue (ten thousand yuan)
landnum	218.6	338.7	107.6	112.8	Construction land area (km2)
n_poll	23.76	51.83	23.38	49.60	The number of polluting firms
boundary	0.378	0.485	0.406	0.492	If the city/county is located in the provincial boundary. Yes = 1, No = 0
region	0.222	0.416	0.125	0.331	If the city/county located in eastern China. Yes = 1, No = 0
shch	0.0792	0.270	0.0313	0.174	If the city/county belongs to a sub-province city. Yes = 1, No = 0
distance	59.84	46.62	61.69	54.35	The distance between counties/cities and their prefecture-level city(km)

Note: authors’ calculation. All the variables above are measured at county level.

**Table 3:** The impact of the CCU policy on air pollution.

VARIABLES	Mean (1)	Mean (2)
pp	3.2709 ***	4.0429 ***
	(2.67)	(3.14)
Observations	28,188	25,799
R-squared	0.9391	0.9415
Controls	No	Yes
County FE	Yes	Yes
Year FE	Yes	Yes
Cluster	County	County

Note: robust t-statistics in parentheses; *** *p* < 0.01, ** *p* < 0.05, * *p* < 0.1.

**Table 4:** Robustness check.

VARIABLES	Mean (1)	Mean (2)	Mean (3)	Mean (4)
pp	3.9190 ***	3.9592 ***	4.4567 ***	4.0429 ***
	(3.06)	(3.31)	(3.64)	(3.17)
Observations	24,020	434	23,815	25,799
R-squared	0.9418	0.9463	0.9426	0.9415
Controls	Yes	Yes	Yes	Yes
County FE	Yes	Yes	Yes	Yes
Year FE	Yes	Yes	Yes	Yes
Cluster	County	County	County	County-Year

Note: robust t-statistics in parentheses; *** *p* < 0.01, ** *p* < 0.05, * *p* < 0.1.

**Table 5:** Spillover effect test.

	Mean		
VARIABLES	Distance ≥ 50 km	Distance ≥ 60 km	Distance ≥ 70 km
pp	4.4270 ***	4.3697 ***	3.0325 ***
	(3.16)	(3.13)	(2.65)
Observations	12,920	10,330	8268
R-squared	0.9390	0.9357	0.9329
Controls	Yes	Yes	Yes
County FE	Yes	Yes	Yes
Year FE	Yes	Yes	Yes
Cluster	County	County	County

Note: robust t-statistics in parentheses; *** *p* < 0.01, ** *p* < 0.05, * *p* < 0.1.

**Table 6:** Mechanism analysis.

	(1)	(2)
VARIABLES	Landnum	n_Poll
pp	165.7036 ***	10.1763 **
	(3.00)	(2.15)
Observations	9403	25,826
R-squared	0.5730	0.7524
Controls	Yes	Yes
County FE	Yes	Yes
Year FE	Yes	Yes
Cluster	County	County

Note: robust t-statistics in parentheses; *** *p* < 0.01, ** *p* < 0.05, * *p* < 0.1.

**Table 7:** The results of the geographical effect.

	(1)	(2)	(3)	(4)
VARIABLES	Boundary	No_ Boundary	Eastern	Midwest
pp	4.7236 *	3.4409 ***	8.2172 ***	4.0253 ***
	(1.88)	(3.22)	(3.44)	(3.40)
Observations	9825	15,961	5282	20,517
R-squared	0.9461	0.9394	0.9557	0.9419
Controls	Yes	Yes	Yes	Yes
County FE	Yes	Yes	Yes	Yes
Year FE	Yes	Yes	Yes	Yes
Cluster	County	County	County	County

Note: robust t-statistics in parentheses; *** *p* < 0.01, ** *p* < 0.05, * *p* < 0.1.

## Data Availability

The data presented in this study are available upon request from the corresponding author. The data are not publicly available due to privacy.
